# What the Vitamine Theories Mean in Practice

**Published:** 1920-06-19

**Authors:** 


### FOOD VALUES AND COMMON-SENSE.

### I. What the Vitamine Theories Mean in Practice.

Up to some eight or ten years ago we were taught to appraise the value of our food in terms of proteins or (tissue builders), fats and carbohydrates (energy and fuel factors), salts of potassium, lime, sodium, etc., and water, and we were given to understand that if we provided a sufficiency of food containing these constituents in proper proportion we had done all that was necessary to maintain life and promote growth. Indeed, as late as 1916 or 1917, when German experts, at the request of their Government, made a report on the food supply of that Empire, this report was entirely compiled in terms of proteins, carbohydrates, and fats, and apparently no account whatever was taken of vitamines, if one may judge by the printed copies received in this country, so that one may excuse the housekeepers of our country if they do not realise the importance of the subject-matter of this article.

### A Necessary Revision.

The first intimation that our ideas on food values required revision came from the discovery that the well-known Eastern disease called beri-beri was caused by the want of some food constituent. This was a rude shock to our preconceived ideas of disease, for we had grown so accustomed to think of disease as caused by a positive agent—a microbe, a toxin, etc.—that our minds could not adjust themselves at once to the idea that, excluding ordinary food insufficiency or famine, disease could be brought about by the absence of a particular constituent in our ordinary food. Beri-beri, a disease that disables many and often leads to death, seemed to be confined to rice-eating populations. Further inquiries elicited the fact that it appeared only to attack those who ate rice ground in modern machinery of steel rollers and polished, and that those who ate rice unmilled or home-milled escaped the disease, and this was confirmed by the occurrence of neuritis in birds fed on this same rice. This discovery naturally led to further inquiry as to what constituent was taken from the rice during the milling process.

It was next found that polished rice had lost the husk or bran, the pericarp or "silver skin" immediately underneath the husk and the germ or portion of the grain which gives rise to a new growth of rice when sown. The next stage was to isolate this mysterious substance contained in the pericarp and germ, and though this attempt has not fully succeeded, an extract was manufactured which can be dissolved in water, and in the course of numerous experiments was found to cure beri-beri when administered to pigeons. This vitamine, therefore, has been called, for want of a more precise knowledge and a better term, the "water-soluble B" or "anti-neuritic factor."

### Scurvy.

The next disease that naturally fell under suspicion as being a deficiency disease was scurvy, and the proofs were easier, for the preventive powers of fresh fruits, fruit juice, and vegetables have been known from almost the earliest times, but could never before be explained, so by means of further experiments the existence of an anti-scorbutic vitamine became firmly established. The last disease which seems to be due mainly to the absence of some constituent in the diet is rickets.

### Rickets.

As is well known, the bones in this disease are deficient in lime salts, and the use of drinking waters from which they were absent or which only contained them in very small amounts were looked upon with grave suspicion. When it was then found that the food of rickety children was apparently *not* deficient in lime salts their deficiency in the bones was then explained by their non-absorption from the food in the alimentary canal in some way. Then came Bland Sutton's experiments in the prevention and cure of rickets in lion cubs by feeding them on cod-liver oil and crushed bones. This first suggested the idea that fat might be the missing constituent, with the consequence that when the existence of vitamines became later on established diligent search showed that for growth in the young and for the prevention of rickets fat was indispensable. Most interesting experiments which prove this beyond a doubt were carried on—recently published by Mrs. Mellanby—and it was further discovered that this vitamine was not the fat itself, since only certain fats possessed the power of promoting normal growth and preventing rickets. At first animal fats were thought to be the only ones containing this vitamine, but in one animal fat at least—viz., lard—it has been found absent, and in one or two vegetable oils it seems to exist in small amounts. From its association and solubility in fats it is therefore known as the "fat soluble A factor."

We have thus discovered up to the present, in all, three food factors—viz., fat soluble A, concerned with growth and the prevention of rickets; water soluble B, also concerned with growth and the prevention of beri-beri; and the anti-scorbutic factor. In the next place someone may argue that these diseases are on the whole very rare in England, and especially beri-beri, which is practically unknown, and scurvy, almost the same; and that therefore these vitamines cannot be so important as physiologists assume, but the reply that nearly all our ordinary food in England contains more or less of these vitamines, in any case sufficient to prevent these diseases, except in a few cases, may seem to some persons to support the argument against bothering about them when they are present everywhere. To that we may reply that in the first case rickets is *not* rare by any means in England, for although we do not often meet the grave deformities formerly associated with the disease, milder forms are extremely common among young children and handicap many of the poorer classes in after life—and the same may be said about infantile scurvy. Further, one has the uneasy suspicion that there are many, at present unexplained, minor ailments, such as certain skin diseases, defective teeth, and enlarged tonsils, adenoids, and numerous other complaints and deviations from normal health, which may be due to deficiencies in these accessory food factors. In a country, therefore, which eats so much preserved food as we do it cannot do any harm if we make some inquiry into those articles of food which lack these accessory factors and guide the housekeeper how to make up deficiencies in some classes of food, by the substitution or addition of other articles which contain vitamines in abundance; and this will be the task of the next article.

